# Community Inclusion Experiences While Establishing Community Mental Health Clubhouses in Taiwan: Perspectives from Mental Health Professionals

**DOI:** 10.3390/healthcare12111067

**Published:** 2024-05-24

**Authors:** Hong Hong, Ching-Teng Yao

**Affiliations:** 1 Bachelor Program of Senior Health Promotion and Care Management for Indigenous People, National Changhua University of Education, Changhua 50007, Taiwan; oscarhong@cc.ncue.edu.tw; 2 Master Program of Long-Term Care in Aging, Kaohsiung Medical University, Kaohsiung 80708, Taiwan

**Keywords:** community mental health clubhouse model, not-in-my-back-yard, community inclusion, mental health worker, qualitative analysis

## Abstract

Community inclusion is a human right for all people, including people with mental illness. It is also an important part of individualized support to enable people experiencing mental illness to live in their preferred communities and to recover. In Taiwan, no study has investigated the social experiences of healthcare professionals. To fill this knowledge gap and inform ongoing mental health system strengthening, this study examined the not-in-my-backyard (NIMBY) phenomenon observed while establishing community mental health clubhouses in Taiwan and corresponding experiences of community inclusion. Data were collected through semi-structured interviews of 16 purposively sampled frontline healthcare professionals from Taiwanese mental health clubhouses. Data were analyzed using qualitative content analysis. Two themes were identified: “NIMBY phenomenon: Community residents’ resistance to clubhouses” and “Measures adopted by the clubhouse for community inclusion”. Two categories with eleven subcategories emerged. The findings demonstrate the following conclusions. First, the NIMBY phenomenon is related to the stigmatization and discrimination faced by individuals with mental disorders in society. Second, in Asian societies, mental disorders are associated with a particular social and cultural context. Third, the fear and rejection of individuals with mental disorders deepen their social exclusion. Forth, community mental health clubhouse models employ seven strategic approaches to community inclusion, listed as follows: capacity building for individuals, direct interaction with the public through community activities, provision of community services, life skills training, repairing and managing neighborhood relationships, assisting individuals in obtaining community employment, and conducting social education for community residents. Clearly, we need to combat social exclusion of people with mental illness and promote inclusive and accessible services and systems across sectors.

## 1. Introduction

In the past 20 years, mental illness has become the third leading cause of the global burden of disease after cardiovascular diseases and cancer [1]. People with mental illness continue to face social exclusion worldwide, which poses challenges to public health and human rights [2]. Taiwan has seen a steady increase in the number of people seeking medical treatment for mental illness over the past decade, from 1.52 million in 2011 to 2.058 million in 2020. However, more than 70% of individuals with mental illnesses do not seek professional assistance [3]. Some harmful incidents involving persons with mental illness have occurred in Taiwan in recent years. Consequently, the Taiwanese government announced the launch of the “Social Safety Phase Scheme (Phase II)” in 2021. The initiative’s objective is to establish a community-based mental health care system for individuals suffering from mental illness [4]. The most recent introduction in community mental health recovery centers is the clubhouse model. This is a community mental health rehabilitation program intended to provide a restorative environment for individuals with severe psychological difficulties. Clubhouses provide members with social, professional, and educational support, assisting them in regaining their psychological well-being. This model is characterized by a collaborative and supportive approach in which members and staff work together to conduct all clubhouse activities to build members’ confidence and capacities for full participation in society. Studies have shown that participation in clubhouse programs can reduce the likelihood of readmission, improve the quality of life and employment prospects, enhance both physical and psychological well-being, and reduce medical resource utilization by individuals with mental illness [5,6,7,8,9,10].

Promoting the social inclusion of people with mental disorders has become an important policy objective worldwide [11,12]. The World Health Organization (WHO) defines social exclusion as a dynamic, multidimensional process driven by unequal power relations. It involves exclusion in four main dimensions—economic, political, social, and cultural—and significantly affects individuals at various levels, including the personal, family, group, community, and national levels [13]. Individuals with mental disorders require policy support to help them integrate into the community and live on an equal basis with others. Thus, social inclusion policies can help individuals with mental disorders feel accepted and reintegrate into society. A social inclusion policy can facilitate the removal of all structural barriers to participation in society and combat societal discrimination against individuals with disabilities [14,15]. Because of societal stigmatization, individuals with mental illness are often perceived as unpredictable, dangerous, and irrational. The prevailing belief in society is that they should be treated in a discriminatory and exclusionary manner [16,17]. Globally, many people with mental illness are excluded from employment (economic exclusion); deprived of their legal rights to vote, marry, or own property (political exclusion); and suffer from social exclusion (socio-cultural exclusion) [18,19,20,21]. The United Nations Convention on the Rights of Persons with Disabilities (CRPD) emphasizes the need for effective and appropriate measures to ensure that all persons with physical and psychological disabilities have the same rights and opportunities to live in the community. This includes efforts focusing on how persons with disabilities can be empowered to live with dignity and equality with others [22]. The mental health policies of developed countries promote employment and housing for people with mental disorders. They are protected by legislation, and anti-discrimination measures are implemented [23]. The following types of policy measures are used: (1) legislation; (2) community support and services; and (3) anti-discrimination measures. In Taiwan, the “Mental Health Act” provides for the care and social inclusion of those with mental disorders, thereby safeguarding their rights. According to Article 3 of the law, the objective is to facilitate and support individuals with mental disorders to live in the community by providing them with the necessary support and assistance for housing, placement, education, employment, upbringing, medical care, social participation, independent living, and other support measures [24]. The clubhouse model is a new community psychiatric rehabilitation policy in Taiwan. These clubhouses provide their members with opportunities for friendship, employment, and education, which help them on their paths towards mental health recovery. Towards that end, the model features clubhouse members and staff working to build a working community by integrating three practice domains: social relationships, unit work, and individuals’ needs and pursuits.

Yet, many community residents oppose the establishment and promotion of community mental health clubhouses. Reasons for the opposition include feeling unsafe about locating these institutions in their communities and their belief that individuals with mental illnesses tend to be violent and perpetrate criminal activity [25]. This resistance increases the difficulty of promoting the community mental health clubhouse model. Stigmatization plays a pivotal role in social exclusion [26]. It acts as both a societal and individual phenomenon. It encompasses personal beliefs, biased attitudes, and discriminatory behaviors [27,28,29]. People often have stigmatized and discriminatory views towards establishing rehabilitation centers for individuals with mental disorders in the community [30,31]. These negative attitudes hinder the social inclusion of those with mental disorders and impede the advancement of mental health policies and initiatives [32]. Therefore, effective social inclusion policies are vital for facilitating the recovery journey of individuals with mental disorders [33].

In Taiwan, following initial resistance in 2003, community mental health recovery centers have attempted to integrate into communities, only to face repeated protests from local residents. This phenomenon is referred to as the NIMBY phenomenon, which describes the emotions, attitudes, thoughts, and actions of individuals or communities opposed to the use of specific land or buildings [34]. Although government officials and bureaucrats believe that the establishment of these centers is not problematic, local communities are strongly opposed to them. Twenty years have passed since the first protest incident involving such a center in Taiwan in 2003. However, even when Taiwan’s mental health policy introduced the new community mental health clubhouse model, community residents still express strong opposition. Evidently, community residents still harbor deep-seated fears and concerns about individuals with mental disorders, who continue to endure social exclusion.

In summary, establishing community mental health clubhouse models faces NIMBYism, while the individuals with mental disorders who actually receive clubhouse services encounter social exclusion. Taiwanese society is currently experiencing significant NIMBYism and continues to demonstrate social exclusion of individuals with mental disorders and mental health recovery centers. Therefore, a more comprehensive exploration of community mental health clubhouses is needed to promote the inclusion of individuals with mental disorders into their communities.

The aim of this qualitative study was to understand psychological health practitioners’ experiences with NIMBY situations while establishing these clubhouses and their opinions on promoting the inclusion of people with mental illnesses in the community. This is essential information for developing programs that promote social inclusion relevant to the local Taiwanese sociocultural context and also for providing a starting point for deeper theoretical consideration on the topic grounded in Asiatic regional knowledge. Overall, this study aims to present new perspectives in current research by engaging with the literature on NIMBY phenomena and social inclusion. This study also seeks to identify concrete recommendations based on practical experience, which can be used as a basis for developing future policy adjustments or services in Taiwan.

## 2. Materials and Methods

This research applied a qualitative methodology. Rooted in symbolic interactionism, qualitative methodology holds that perspectives and human actions are fundamentally influenced by contexts and social processes, and this method is therefore suitable for studying staff members’ practices in the clubhouse environment. This study specifically focused on frontline mental health practitioners’ perspectives and examined how frontline professionals manage the atmosphere of community resistance and opposition from residents when establishing community mental health clubhouses. It also explored how they conduct community inclusion work, engage in social education with residents, and promote the inclusion of individuals with mental disorders into the community.

### 2.1. Participants

Participants were primarily selected based on the study’s purpose. First, guided by the research objectives and questions, participants who met the following criterion were recruited: current mental health professionals working in clubhouse model rehabilitation institutions (nurses, social workers, psychologists) with at least one year of work experience at the institution. Second, a purposive sampling method was used. More participants were selected based on referrals from the initial interviewees to increase the richness and diversity of the study. Expanding the scope of the study’s interviews allowed for the coverage of a wider array of service facilities and various forms of community inclusion across different areas. Third, participants were required to be frontline professionals actively involved in planning and implementing community inclusion services, as well as be willing to participate in in-depth interviews throughout the study. Each participant was informed of the study’s purpose and signed a consent form in accordance with academic ethical standards. This study was conducted from October to December 2023. A total of 16 frontline mental health practitioners working in community mental health clubhouse models were interviewed.

### 2.2. Research Ethics

This study was approved by the Institutional Review Board and was conducted with the consent of the institution. The researcher explained the study’s objective and method to the participants and acquired their written consent. Their anonymity and confidentiality were strictly protected. All data were encoded to ensure the anonymity of the participants and used only for academic research purposes. The participants were permitted to withdraw from a session or quit the study altogether at any stage for any reason.

### 2.3. Data Collection

This study primarily employed semi-structured, in-depth interviews. An interview outline was emailed to the participants prior to the interviews and included several sections, as follows: NIMBY situations encountered in the process of establishing community mental health clubhouses, actual practices of community inclusion adopted by the clubhouse, and acceptance of individuals with mental disorders by community residents after the implementing of these practices. The interview locations and times were selected considering the convenience of the interviewees, with most interviews happening in the units where they worked. The interviews lasted between 60 and 90 min and were fully recorded with the interviewee’s consent. Two face-to-face interviews were conducted with all participants. Participants also voluntarily provided substantial information regarding the practical approaches to community inclusion within their institutions. This included records of interactions between the institutions and community residents, activities organized by the institutions to promote community inclusion, and official correspondence, such as letters and documents. These data serve as important background information for understanding the interview content and can be used for cross-referencing during data analysis.

### 2.4. Data Analysis

The collected data were analyzed using the qualitative content analysis method proposed by Graneheim and Lundman [35]. The following steps were involved: (1) multiple readings of the transcripts to understand the overall content; (2) reading and coding the text data, and continuously comparing and contrasting it to understand its meanings and relationships therein; (3) inductive analysis of the data to identify common themes and classifying data with similar meanings to develop core and sub-categories; and (4) analyzing the meaning, rules, and concepts from the collected data to arrive at the research findings.

### 2.5. Rigor

This study’s rigor and trustworthiness were examined using the following four criteria proposed by Guba and Lincoln on the precision of qualitative research [19]: (1) Credibility: The researchers had extensive experience in studying community-based mental health policies and were well acquainted with the process of promoting mental health policies in Taiwan. Furthermore, the researchers had received comprehensive training in qualitative research, demonstrating practical proficiency in conducting interviews and performing qualitative analysis. In addition, regular discussions with qualitative research experts were an integral part of the research process. (2) Transferability: Interviews were accurately and truthfully transcribed verbatim for presentation. The transcriptions of interviews were returned to participants for correction. (3) Dependability: Two community mental health professionals with extensive experience in qualitative research were invited to review and modify the classification of the findings. (4) Conformability: The researchers safeguarded all reflective field notes and records of data analysis for future verification and reference. In the final stage, the participants were given the opportunity to review and confirm the research outcomes.

## 3. Results

The 16 frontline professionals interviewed included 10 females and 6 males. The average work experience was 5 years. Social work was the most common professional background among the participants, followed by nursing. Most institutions were privately owned and operated, and the majority were located in northern Taiwan (Table 1).

The interview data were analyzed and are presented in two main parts. First, an analysis of the NIMBY phenomenon is presented, revealing four categories of resistance to the clubhouse model within the community. Second, the strategies institutions had implemented in response to community resistance in seven different categories are shown (Table 2).

### 3.1. Analysis of the NIMBY Phenomenon of Community Resistance to the Clubhouse Model

#### 3.1.1. Biased Impressions and Stereotypes

The clubhouse model was opposed by the community from the outset, with residents petitioning to expel it. Upon learning of the clubhouse’s planned establishment in the community, residents protested by hanging banners, expressing concerns about falling property prices and the diminished quality of life. In some cases, residents even petitioned their representatives, arguing that individuals suffering from mental disorders would pose a security risk in the community, particularly to schools, and that they should be kept out of the community. This relates to the negative stereotypes and biased initial impressions that Taiwanese people have formed about individuals with mental disorders. Some statements from participants are noted below:

*The reasons for the community residents’ opposition are related to their negative stereotypes about individuals with mental disorders. Their concern is that such centers will affect their safety, people with mental illnesses will create disturbances, and property values would decrease. Stereotypes are to blame for all of these.*

(F2)

*It’s just that the community residents have a negative stereotype about our kind of institution… They say we’re trouble-seekers and will cause a lot of problems for the community, calling us “time bombs” behind our backs… It’s a stereotype that residents have.*

(F7)

*These individuals with mental illness are too dangerous! They should be isolated in hospitals. How can they be released from hospitals and come to the community? There are elderly people and children in the community. It’s very dangerous. It’s just a lot of these stereotypical imaginations!*

(F10)

*They say that in TV, news reports portray individuals with mental illness like they are always attacking people! … Aside from themselves, their families and their children would all be anxious and afraid! This has led to a lot of negative thinking…*

(F13)

#### 3.1.2. Social Stigma and Exclusion and Being Labeled as Dangerous

Several incidents of random murder involving individuals with unstable mental health conditions have occurred in Taiwan in recent years, which have been accompanied by extensive media coverage. This has contributed to the public perception that individuals with mental illness are dangerous. Many people attribute unrelated incidents in the community to individuals with mental illnesses. Some participants noted the following:

*News media often report negative news about individuals with mental illness randomly attacking others. Over time, this has led to serious stigma and exclusion of people suffering from mental illness in society, who were all tarred with the same brush, and all individuals with mental illness have been labeled as dangerous.*

(F6)

*Whenever something bad happens in the community, they blame it on our mental health patients! They see mental health patients as dangerous individuals, believing that the increase in negative incidents in the community is due to the establishment of our mental health recovery center here, which is discrimination against them.*

(F9)

*When they see us, they say we’re a place for lunatics, stigmatizing us in many ways. For example, they say that people with mental illness are uncontrollable and dangerous to the community. They blame many bad things that happen in the community on us, like defecation and urination in the community park, but later it’s always proven not to be our patients.*

(F15)

#### 3.1.3. Fierce Resident Protests: Reflection of Fear of Individuals with Mental Illness

According to participants, community residents acknowledged that their fear of individuals with mental illness reflects a common societal value system. Residents’ strong opposition only arose because the center would be located in their community. A few participants noted the following:

*Approximately 40% of the residents in the community express opposition and vehement protests (such as hanging banners and throwing eggs). In fact, many residents living in this community have a relatively high socioeconomic status. They are concerned that having a mental health recovery center in their community can make people worry about whether patients in your center will steal things or engage in violent behavior. Every day, various individuals with mental health issues come to our area, causing our community to feel unsafe.*

(F3)

*The community residents are disdainful about having individuals with mental disorders for neighbors, which is a serious form of exclusion. With the arrival of this group in the community, residents harbor significant fear and apprehension, worrying that the community will become markedly unsafe.*

(F8)

#### 3.1.4. Residents’ Thoughts Rife with Irrational Beliefs

Some residents may not directly express their concerns or understand the underlying reasons for their deep-seated fears. Hence, they may raise various objections and doubts. Some concerns are relatively straightforward to verify and clarify with concrete evidence. However, assuaging or clarifying many other concerns is challenging. Two participants noted the following:

*I don’t know if these individuals with mental illness are stable. Our community used to be a quiet place, and you suddenly coming in to establish such a center has brought about significant changes to our previously peaceful living environment. They should return to the hospital instead of coming to the community and making it out to be a time bomb, making the whole community feel unsafe and causing anxiety among people.*

(F4)

*Usually, I also donate to social welfare organizations, but is it good to place individuals with mental illness in the community? Don’t say we lack compassion. I feel that most individuals with mental illness are unstable. By placing this center in our community, it makes the community residents feel unsafe, and we have to be very careful when leaving our homes.*

(F16)

### 3.2. Analysis of Community Inclusion Efforts Undertaken by Mental Health Recovery Clubhouse Models

Participants’ experiences revealed seven types of community inclusion strategies employed by community mental health clubhouse models in response to the NIMBYism of community residents, listed as follows: providing labor-based community services, organizing or participating in exchange activities, offering independent life skills training for patients, facilitating the establishment and maintenance of friendships, managing neighborhood relationships, assisting individuals with mental illness in finding community employment, and inviting outsiders to come to the center or introducing the center to the outside community.

#### 3.2.1. Empowering Individuals through Capacity Building

Empowerment refers to the idea that individuals with mental disorders can overcome their illness through active effort and strong social support, even while coping with their illness. Through it, they learn that the illness is a part of their life and not the entirety of it. Consequently, they can recognize their strengths and assume various roles and responsibilities within their families and communities. They can harness their abilities, discover their strengths, and reestablish new meanings, goals, and hopes in life. Some participants stated, as follows:

*The clubhouse encourages peer support between individuals, allowing them to listen and see how others perceive their illness, which helps them change their perspectives. In the past, they may have felt burdened by their illness and misunderstood. However, by listening to others’ experiences, they can learn how to cope with their situation.*

(F6)

*Through their inner exploration, they seek ways to recover better or unleash their potential. Individuals share and discuss with each other, gaining a better understanding of the causes of their illness. They continue to consider ways to cope with the illness, exchanging experiences and conditions with one another.*

(F11)

#### 3.2.2. Providing Community Services

Community service is one of the community inclusion methods frequently mentioned by participants and recognized by all participants as a good way to build positive relationships with community residents. Notably, the content of community services is primarily labor-intensive, with cleaning and recycling being the most common. Institutions determine which projects are suitable based on their location and available resources. A few participants noted the following:

*Our center is located inside a community building. To help the residents of the building better understand our patients, we arrange for them to be in contact with our patients on a daily basis. For example, we take on labor-intensive tasks such as cleaning the underground parking lot in the building.*

(F1)

*The recycling center is where the community’s recyclables are collected. Most residents in this community are shop owners. For example, in order to try and foster harmony with the neighbors, we go into these shops owned by the residents and help them pull out the items to be recycled using a cart. We have to pull several carts a week, even from several shops… It’s hard work, but we have been doing it for many years.*

(F8)

#### 3.2.3. Direct Dialogue with the Public through Community Activities

The clubhouse patients directly engage with the public through activities such as “the human library” and large-scale community events. They reflect on themselves and reorganize their thoughts based on the audience’s feedback. Simultaneously, these activities allow residents to view individuals with mental disorders from different perspectives. Some participants stated the following:

*During activities where patients share their life stories, residents often find them captivating. They offer encouragement and feedback, remarking, “You don’t seem to be affected by your previous illness anymore. Keep it up!”. Residents express that the experience is different from their preconceptions about individuals with mental illness, leading to a shift in their perspectives. This change in perception can be considered a success. Additionally, some residents share that their friends have had similar experiences.*

(F5)

*The community residents who engage in the activities often express their astonishment, asking questions like, “How did you endure such a tragic event? We can hardly believe it. How did you find the strength to overcome it?”. Now, seeing you so lively, just like a normal person, how did you come out of it? They feel that you are incredibly amazing, and your image is different from their original impression of people with mental disorders…*

(F7)

#### 3.2.4. Providing Life Skills Training

The community inclusion of inclusion with mental disorders ultimately aims to reintegrate them into society. However, due to the impact of the disorders, they may exhibit social withdrawal. To achieve community inclusion, the clubhouse often combines life skills training with community activities to train the clubhouse residents in independent living skills and promote interaction with community members. Some participants noted the following:

*After treatment and life skills training, they can regain good independence in daily living. However, more importantly, they can integrate into the community. Therefore, life skills training is an important factor in helping them integrate into the community.*

(F8)

*We will train them through life skills training on how to interact with community residents. When they interact with community residents, if there are inappropriate remarks or behaviors, I will wait until they come into the clubhouse, and then I will talk to them about those inappropriate behaviors. When we talk to community residents, we should pay attention to those things…*

(F12)

#### 3.2.5. Repairing and Managing Community Neighbor Relations

If a community mental health clubhouse has faced protests from community residents during its establishment, individuals with mental disorders may easily sense social exclusion in the community. Therefore, facilitating their acceptance by community residents, and repairing and managing community relationships becomes an important task for the facility to promote the integration of individuals with mental disorders into the community. Two participants described the following:

*Initially, we crafted around a thousand small gifts, which we distributed within the community. We emphasized that these were all handmade by our patients.*

(F7)

*We encourage them to leave the clubhouse, for example, to go to nearby stores to shop. Throughout the shopping process, they can engage in conversations and interact with residents, fostering lasting relationships with them.*

(F13)

#### 3.2.6. Assisting Patients in Community Employment

As a primary approach, community inclusion research consistently highlights assisting socially excluded individuals with mental disorders in entering the job market. Employment not only provides patients with a direct income source but also helps expand their social support network within the community. Study participants indicated that providing long-term support and encouragement for individuals to obtain community employment is crucial to help them maintain stability in their jobs within the community. A few participants noted the following:

*Counselors find jobs for patients through visits to the community. After that, well-trained individuals are recommended to the job market. Residents get to know them better through employment, so their perception of them improves.*

(F10)

*We encourage them to leave the clubhouse and seek job opportunities in the community. During this process, residents gradually get to know them. While working, we have counselors to assist them. They help them deal with work-related stress or difficulties in interacting with colleagues, and help them integrate into the community more quickly.*

(F14)

#### 3.2.7. Conducting Social Education for Community Residents

An essential strategy for promoting community inclusion involves using social education to address general public discrimination and stigma against individuals with mental illness. This effort aims to increase the understanding, acceptance, and support for people with mental disorders, while establishing a welcoming and supportive environment for them. Some participants stated, as follows:

*In some situations, when we need to interact with residents or collaborate with community organizations, we hope to help them understand mental health patients. For example, we provide courses on understanding mental illness and how to interact with people with mental disorders. We incorporate these concepts into our courses.*

(F2)

*Educating the public is crucial for fostering community inclusion. It’s important to ensure that the general public understands that our patients are stable, highly functional individuals who are capable of working just like anyone else.*

(F9)

## 4. Discussion

A crucial important task of community mental health recovery centers is promoting community inclusion. However, little attention has been paid to this issue in Taiwan. This study interviewed 16 participants involved in clubhouse models to explore their experiences in fostering the community inclusion of individuals with mental disorders. The goal of this study was to advance the well-being and rights of individuals grappling with mental disorders in Taiwan. This study’s results indicate that community mental health recovery facilities in Taiwan face significant NIMBYism during their establishment because of social exclusion. According to Walker (2017), social exclusion refers to the exclusion of members of society from various aspects of society, including social, economic, political, and cultural dimensions. Conversely, a person’s integration into society is also determined by these systems [36].

First, the main reason for NIMBYism towards Taiwanese mental health recovery facilities is the stigma and discrimination prevalent among the general public. A common misconception is that individuals with mental illness lack autonomy and pose a threat to personal safety when living in the community. This misunderstanding is exacerbated by the media’s extensive negative coverage of cases involving individuals with mental illness. In Taiwan and Hong Kong, incidents of individuals with mental illness committing random acts of violence may contribute to the public’s perception of them being violent offenders [37]. Furthermore, the Taiwanese media frequently uses the term “time bomb” to describe individuals with mental illness, further stigmatizing them. Second, mental illness is often explained by cultural and social contexts in Asian societies. The psychological issues of individuals are frequently attributed to their own weaknesses or societal and cultural influences. Moreover, some believe that mental illness is primarily caused by the influence of evil spirits or karmic retribution from previous lives. Thus, mental illness is perceived as a rare and incurable condition, causing public fear and discrimination. Third, individuals with mental disabilities are further excluded in society due to their stigmatization and negative perceptions. Therefore, opponents argue against the construction of community mental health centers in their “backyards” [38,39,40], contending that the government should handle individuals with mental disorders via medical isolation. They are concerned that establishing such facilities in their neighborhoods will compromise their personal security and negatively impact community life [41].

Fourth, this study reveals the following seven community inclusion strategies adopted by Taiwan’s community mental health clubhouses: empowering patients with skills and knowledge, engaging in direct interaction with the general public through community activities, providing community services, offering life skills training, repairing and managing community neighborly relationships, assisting patients in obtaining community employment, and conducting social education for community residents. Notably, contemporary perspectives on disabilities emphasize barriers within the social environment rather than on the impairments experienced by the individual themselves. Indeed, the International Classification of Functioning, Disability, and Health and the CRPD recognize disability as the complex interaction between an individual’s health condition, environmental factors, and personal factors [22,42]. Negative attitudes in the social environment can thus hinder the effective participation of individuals with disabilities in society. Therefore, when individuals with disabilities face social exclusion, adopting a positive and proactive mindset is crucial. Mental health professionals should take the first step in community inclusion by empowering patients through capacity building and actively assisting them in overcoming these community obstacles [43,44,45].

Moreover, the general public’s negative perceptions of individuals with mental illness are based on social constructions. In certain countries, such as Taiwan, these individuals with mental illness have long been perceived as threats to societal order. This is a barrier to their social inclusion. Conversing with the general public can address public misconceptions and foster their understanding of individuals with mental illness. Countries like New Zealand, the United States, and Canada emphasize social education to enhance public awareness of mental illness and reduce discrimination [46,47,48]. Several strategies are being employed to eliminate discrimination and fear of individuals with psychological health issues, including mass media campaigns, public engagement, and promoting public understanding. Several Asian countries, including Singapore, Japan, and South Korea, are attempting to reduce discrimination against mental health patients by promoting psychological well-being awareness among the public [49,50,51,52]. Furthermore, there has been strong emphasis on actively guiding the community so that residents can better understand and accept individuals with mental illness [53]. Another important community inclusion strategy for individuals with mental disabilities is community support and services. These aim to enable them to effectively participate in social activities, seek employment rights on an equal basis with others, and establish meaningful relationships recognized by others. This study reveals that by providing life skills training and assisting patients with community employment, the clubhouse model emphasizes person-centered and recovery-oriented interventions. These can effectively help individuals with mental disabilities reintegrate into society and assume meaningful roles and relationships. Therefore, community-based rehabilitation is designed to assist individuals with mental disabilities in integrating into society by providing employment services in the community. They are actively assisted so that they can contribute to their communities. Furthermore, community members are encouraged to value the participation of individuals with mental disabilities in community activities and respect their human rights.

Fifth, many countries use legislative measures to promote the integration of individuals with mental disabilities into society. A prime example is legislation that ensures employment opportunities for individuals with mental disabilities and prohibits employment discrimination by requiring employers to make job arrangements based on the condition of those with mental disabilities. Taiwan, for example, has passed laws such as the “People with Disabilities Rights Protection Act” and “Mental Health Act,” which are specifically intended to benefit people with disabilities. These legal mechanisms aim to protect the social inclusion rights of individuals with mental disabilities and reduce employment discrimination in society. Yet, research has demonstrated that while legislation is an important tool, it alone is not sufficient to promote the social inclusion of individuals with mental disabilities.

This research has several limitations. First, purposive sampling was used, which limits the external validity of this study. Second, the study participants only included practitioners from mental health clubhouses. Their viewpoints may not necessarily align with those of policymakers and expert scholars. Hence, future research should also encompass these stakeholders, encouraging a three-way dialogue involving academia, government, and the industry. Third, the sample size was limited. Future works should explore the community resistance to and inclusion status of different mental health clubhouses. Fourth, this study relied on qualitative research methods, interviewing front-line mental health workers who have managed and handled community inclusion in service agencies for individuals suffering from mental health challenges in Taiwan. Scholars can explore the use of quantitative research surveys to collect feedback from the general public, thereby providing an understanding of the public’s perceptions of mental health clubhouses. Also, this study of clubhouse practices may support the development of future research and practice models on how practitioners can intentionally interact with people with severe and persistent mental illnesses to promote recovery.

## 5. Conclusions

The CRPD explicitly states that individuals with disabilities, including those with mental health conditions, should enjoy the same rights to live in a community and make the same choices as other individuals. Therefore, countries should take effective and appropriate measures to ensure that individuals with mental health disabilities can exercise this right and participate fully in their communities. However, this study’s results show that the community mental health clubhouse model still faces stigma and discrimination towards individuals with mental health challenges. The NIMBY attitude towards the clubhouse is rooted in various irrational beliefs, prejudices, and fears that surround individuals who suffer from mental illness. Planners and implementers of community inclusion for clubhouses continue to develop strategies to achieve community inclusion. Some strategies include engaging in direct communication with the public through community activities, providing community services, providing life skills training, repairing and managing neighborhood relationships, assisting concerned individuals with employment in the community, and providing social education to the community. Encouragingly, the community inclusion of individuals with mental disorders has become increasingly important worldwide. The process of community inclusion is, however, a complex one. We need continued exploration, as well as an understanding of the connections that community mental health clubhouses engender among their clients, service providers, and residents of the community.

## Figures and Tables

**Table 1:** Demographic characteristics of participants (N = 16).

Characteristics	Categories	N	%
Age (Years)	20–29	4	25.0
	30–39	5	31.2
	40–49	7	43.8
Gender	Male	6	37.5
	Female	10	62.5
Professional background	Social work	9	56.3
	Nursing	5	31.2
	Psychology	2	12.5
Job tenure (Experience)	1–3	2	12.5
	4–6	8	50.0
	7–9	6	37.5
Organizational attributes	Private	10	62.5
	Public	2	12.5
	Government-owned and civilian-run	4	25.0
Geographical distribution	North	7	43.8
	Middle	4	25.0
	South	3	18.7
	East	2	12.5

**Table 2:** Summary of themes and subthemes emerging from the interviews.

Theme	Subtheme
1. Analysis of the NIMBY phenomenon of community resistance to the clubhouse model	1.1 Biased impressions and stereotypes
	1.2 Social stigma and exclusion and being labeled as dangerous
	1.3 Fierce resident protests—reflection of fear of individuals with mental illness
	1.4 Residents’ thoughts rife with irrational beliefs
2. Analysis of community inclusion efforts undertaken by mental health recovery clubhouse models	2.1 Empowering individuals through capacity building
	2.2 Providing community services
	2.3 Direct dialogue with the public through community activities
	2.4 Providing life skills training
	2.5 Repairing and managing community neighbor relations
	2.6 Assisting patients in community employment
	2.7 Conducting social education for community residents

## Data Availability

The data presented in this study are available upon request from the corresponding author.
